# A State of the Art on Cryogenic Cooling and Its Applications in the Machining of Difficult-to-Machine Alloys

**DOI:** 10.3390/ma17092057

**Published:** 2024-04-27

**Authors:** Mehmet Erdi Korkmaz, Munish Kumar Gupta

**Affiliations:** 1 Department of Mechanical Engineering, Karabük University, Karabük 78000, Turkey; merdikorkmaz@karabuk.edu.tr; 2 Faculty of Mechanical Engineering, Opole University of Technology, 76 Proszkowska Str., 45-758 Opole, Poland; 3 Department of Mechanical Engineering, Graphic Era (Deemed to be University), Dehradun 248002, India

**Keywords:** titanium, inconel, surface roughness, cryogenic cooling, machining

## Abstract

Cryogenic cooling has gathered significant attention in the manufacturing industry. There are inherent difficulties in machining materials that are difficult to machine because of high levels of hardness, abrasiveness, and heat conductivity. Increased tool wear, diminished surface finish, and reduced machining efficiency are the results of these problems, and traditional cooling solutions are insufficient to resolve them. The application of cryogenic cooling involves the use of extremely low temperatures, typically achieved by employing liquid nitrogen or other cryogenic fluids. This study reviews the current state of cryogenic cooling technology and its use in machining difficult-to-machine materials. In addition, this review encompasses a thorough examination of cryogenic cooling techniques, including their principles, mechanisms, and effects on machining performance. The recent literature was used to discuss difficult-to-machine materials and their machining properties. The role of cryogenic cooling in machining difficult materials was then discussed. Finally, the latest technologies and methods involved in cryogenic cooling condition were discussed in detail. The outcome demonstrated that the exploration of cryogenic cooling methods has gained prominence in the manufacturing industry due to their potential to address challenges associated with the machining of exotic alloys.

## 1. Introduction

**Figure 1:**
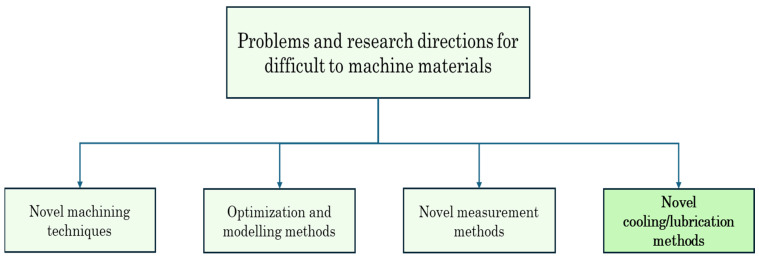
The problems and research directions for difficult-to-machine materials.

A significant part of every economy is the manufacturing sector, which uses a wide variety of production techniques [1]. One of the methods used in the production of any workpiece is the machining method [2]. The machining method is based on the principle of cutting the excess parts by removing the chip in order to obtain the desired shape on the raw workpiece in the desired size, tolerance, and quality [3,4]. The superior features of the machining method, such as its ability to be easily applied to different materials [5], its ability to produce tolerances close to exact measurement [6], and its ability to easily process different geometries with diversified machine-tool equipment, have provided it with advantages over other manufacturing methods [7]. Performing the machining method with high efficiency is of great importance in terms of product costs and performance [8]. It is desired that the cost of items, such as energy consumed during operation and cutting tool life, are at low levels, and product features such as surface quality and dimensional accuracy are at high levels [9,10]. To achieve optimum efficiency in machining, it is crucial to accurately estimate the machining and cutting tool parameters based on the workpiece material, and to decide on the suitable machining parameters accordingly [11]. It is a common practice to use cooling applications to increase efficiency and solve problems encountered in machining methods [12,13]. Cooling applications within the scope of machining have been used since the mid-19th century [14]. The high heat released during machining negatively affects operational safety, especially cutting tool performance [15,16]. Effective removal of this high heat generated during cutting from the cutting area is also possible with an effective cooling application [17]. Effective cooling removes the heat generated in the cutting area, accelerates chip evacuation, creates a film layer between the tool and chip, lubricates and reduces friction [18]. With all these contributions, cooling application increases tool life and operational efficiency [19]. However, the use of chemical-based cooling fluids causes harm to human health and environmental pollution problems [20]. If waste management of chemical-based coolants is not done effectively, it causes great harm to the environment [21]. This situation makes environmentally friendly approaches in cooling applications and research on achieving high efficiency with low amount of refrigerant usage important [22]. These approaches and the needs of machining methods have led to the formation of different types of cooling applications [23,24]. Based on operational needs, conventional cooling, MQL, and cryogenic cooling have been developed [25]. High efficiency in machining is possible by reducing costs without compromising product quality. Therefore, tooling costs [26], energy consumption [27], and cooling costs [28] must be kept low. Determining appropriate cutting parameters besides cooling conditions in terms of operational costs is among the objectives of the mentioned studies [29,30,31,32]. Figure 1 shows the problems and research directions for difficult-to-machine materials.

Therefore, this review article focuses on the most recent topic in these lines, which is known as new innovations and changes in cryogenic cooling. These modifications include the incorporation of nanoparticle-added nanofluids and/or minimum quantity lubrication.

## 2. Difficult-to-Machine Materials

Materials that are difficult-to-machine include alloys based on steel, nickel, and titanium [33,34,35,36]. In general, “Difficult-to-machine materials” are named as such due to their high strength, high ductility, low thermal conductivity, high susceptibility to strain, high strain rates, and hardening [37]. The low thermal conductivity of difficult-to-machine materials leads to heat concentration in the cutting zone [38]. As a result, machining these types of materials negatively affects properties such as high residual stresses [39], high cutting forces [20], high cutting temperature [39], rapid tool wear [40], accumulation of chip (BUE) formation in the cutting tool [41], surface quality [42], fatigue life [43], and corrosion resistance caused by chip-breaking difficulties [44]. It is vital to consider the heat generated by the cutting tool and the workpiece, as it relates to machining mechanics and tool life. Almost all of the energy spent during machining turns into heat in the cutting zone, which is important in terms of tool life, cutting forces, chip form, and workpiece surface quality [45,46,47]. Despite the trend toward dry cutting, cutting fluids are preferable for machining these materials because dry cutting reduces product quality and tool life [48]. The dimensional accuracy of materials that cause rapid tool wear and are difficult-to-machine is primarily due to tool wear [49]. Additionally, if the wear on the rake surface of the cutting tool exceeds a critical point, chip breakability may also pose a problem in terms of machinability [50]. Cooling and lubricating strategies have been used to improve machinability in difficult-to-machine materials due to abrasive tool wear, surface quality degradation, and high cutting temperatures [51,52]. Application of cooling in the cutting process can increase tool life [53] and dimensional accuracy [54], reduce cutting temperature [55], improve productivity by reducing surface roughness [56] and the amount of power consumed for the process [57]. However, cutting fluids are not only harmful to the environment, but are also dangerous to health [58]. These issues have led to several attempts to reduce or eliminate cutting fluid [59,60].

### 2.1. Machining Characteristics of Difficult-to-Machine Materials

#### 2.1.1. Tool Wear

According to machining research, friction, pressures, and high temperatures at the tool-chip and work-piece-tool interface cause elastic and plastic deformation and tool wear [61,62,63]. Shape changes and wear that occur in the tool during machining negatively affect the machining process [64]. The negative effects caused by wear on the tool are the decrease in tool life [65] and, accordingly, the increase in production costs and the decrease in process quality [66]. In order to accurately determine tool life, it is necessary to know the tool failure mechanisms that negatively affect tool life and the reasons for these mechanisms [67]. Cutting tools end their life through wear, plastic deformation, or breakage. An ideal cutting tool should have the following features listed below [68,69,70]:

- High temperature hardness for operation at high temperatures.
- High elastic and plastic deformation resistance against high tension.
- High fracture toughness against impacts occurring during machining.
- Chemical stability, especially at high temperatures, for resistance to diffusion and chemical and oxidation corrosion.
- High thermal conductivity to prevent heat accumulation on the tool cutting edge,
- High fatigue resistance in intermittent machining.
- High thermal shock resistance against heating and cooling that occurs during cutting.
- High rigidity for dimensional stability.
- Suitable friction properties to prevent the formation of chip accumulation (Built-up Edge-BUE), especially in the machining of soft, ductile materials.

**Figure 2:**
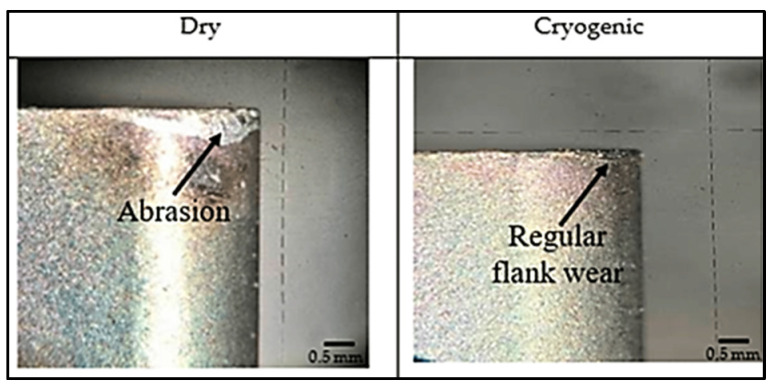
Mechanism of tool wear under different conditions [71].

The effective wear mechanism in free surface wear is the abrasion mechanism as shown in Figure 2. For these reasons, the heat generated in the contact area has a great effect on tool wear, and therefore the importance of cryogenic cooling is increased [71].

#### 2.1.2. Surface Roughness

**Figure 3:**
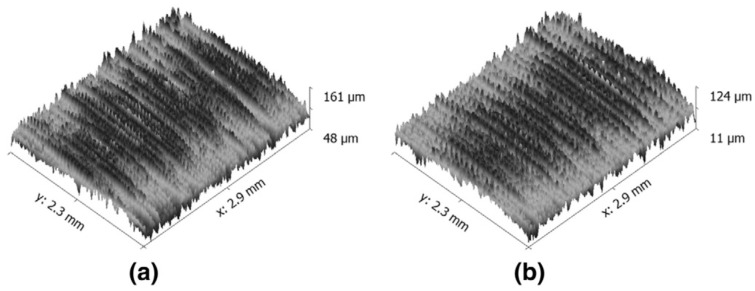
Surface roughness variations in terms of dry and cryogenic cooling conditions (**a**) Dry and (**b**) Cryogenic conditions [79].

Due to physical, chemical, thermal, and mechanical movements between the cutting tool and the workpiece, undesirable machining marks appear on machined surfaces depending on the machining method, cutting tool, and conditions [72,73,74]. Surface roughness is the situation that creates irregular deviations above and below the nominal surface line [75,76]. It is inevitable for surface roughness to occur on machined surfaces, regardless of the work material and machining method used [77]. A key quality consequence in machining is part surface integrity [78]. Figure 3 shows the surface quality under dry and cryogenic machined surfaces.

#### 2.1.3. Cutting Temperature

The plastic deformation on the workpiece and chip flow from the cutting tool surface generate heat during machining processes. The heat generated on the surface of the cutting tool when the cutting tool contacts the workpiece is called the cutting tool temperature [80]. The distribution of the resulting temperature is related to heat loss due to thermal conductivity, heat capacity, radiation, and convection of the workpiece and cutting tool. Since most of the power spent in machining processes turns into heat, cutting forces, tool wear, residual stresses, and many other features are seriously affected [81]. There are three basic heat generating sources during the machining process [82].

- Primary shear zone (PSZ): The area where the majority of the material is removed or deformed during cutting is called the primary shear zone in machining. In this region, the cutting tool exerts the forces that ultimately lead to the shearing of the workpiece [83].
- Secondary shear zone (SSZ): As the chip undergoes plastic deformation for the second time, heat is generated, and less heat, but a higher temperature than PSZ, is absorbed by the workpiece [84].
- Tertiary shear zone (TSZ): The friction of the cutting tool’s side surface on the workpiece’s machined surface generates heat in this region, and wear on the side surface enhances it. The minimum heat generated is absorbed by the cutting tool [85].

**Figure 4:**
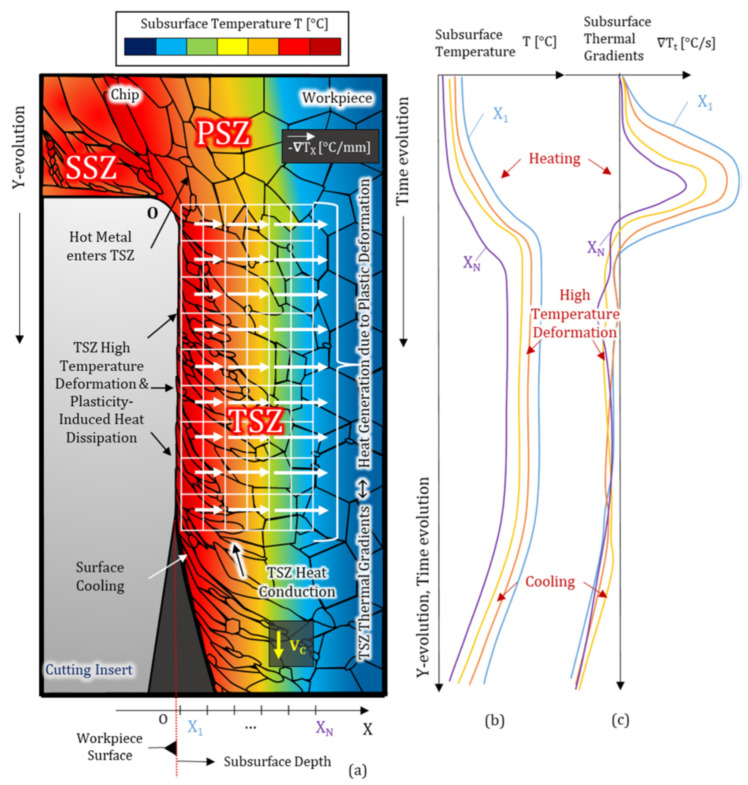
The heat production and distribution in the machining process (**a**) Subsurface thermal field and spatial thermal gradients induced to the machined surface by the plastic deformation process; (**b**) temperature and (**c**) thermal gradients time evolution at different subsurface depths as the workpiece layers cross the tool-workpiece interface, showing heating and cooling phases separated by a high-temperature deformation region [82].

In other words, as long as the cutting process continues, heat production continues and is distributed among the cutting tool, workpiece, chip, and cooling/lubricating fluid. In Figure 4, heat production and distribution in the machining process are expressed in detail on the two-dimensional model.

**Figure 5:**
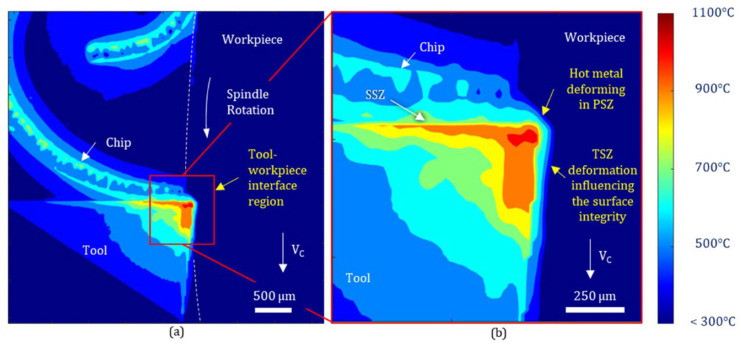
The geometrical association of a temperature field in a cutting tool based on heat generation zones (**a**) Representative characteristics of the cutting-induced thermal field at the tool-workpiece interface. (**b**) Magnified view at the tool tip area to enable identification of metal shear zones (i.e., PSZ, SSZ and TSZ) [82].

**Figure 6:**
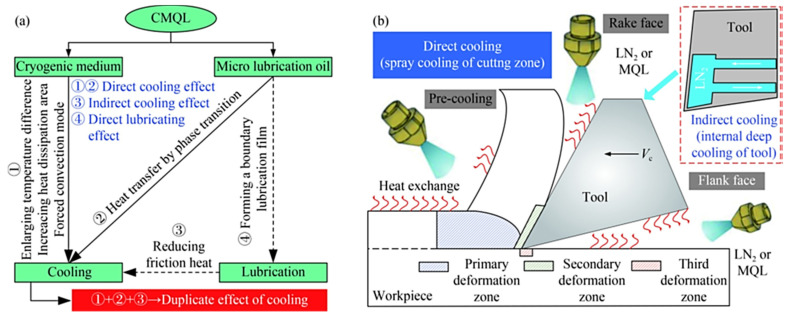
The CMQL cooling mechanism and methods for enhancing its cooling impact are superimposed: (**a**) four distinct types of cutting temperature influence, (**b**) various jet positions combined [86].

Cutting temperature varies by tool, chip, and workpiece. In Figure 5, the highest temperature is on a line away from the cutting edge. By optimizing tool geometry, machining parameters, and coolant, machining temperature can be reduced. Figure 6 shows that cooling fluids are the best way to lower cutting temperatures.

#### 2.1.4. Cutting Forces and Power Consumption

**Figure 7:**
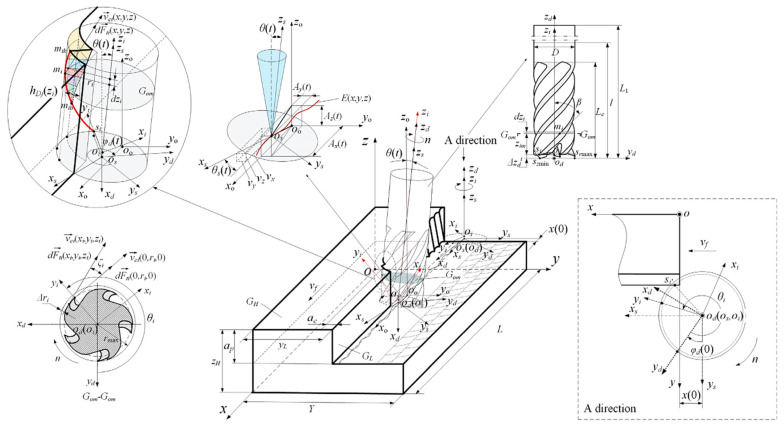
Energy consumption model from main cutting force in a milling operation [87].

In machining, cutting forces directly impact heat generation, tool wear, surface quality after machining, and dimensions of the workpiece. Main cutting force (Fc), feed force (Ff), passive radial force (Fp), and resultant force (Fr) are the four forms of cutting forces used in machining processes (Figure 7).

Machining difficult-to-machine materials reportedly results in high cutting forces, which in turn leads to increased tool wear [88]. There is a strong relationship between the power required for metal removal and cutting forces [89,90]. The power consumed in the cutting process is the factor that determines energy consumption, which is one of the most important cost factors in production. Combining the cutting speed (V) with the main cutting force (Fc) yields the necessary power (Pc) for machining. Cutting depth (ap), feed rate (f), and the material’s particular cutting resistance (kc) component all contribute to the main cutting force’s magnitude [91]. The main cutting force (Fc) is the most important parameter that determines the power spent on machining and therefore the energy cost [92]. Cutting forces are an important parameter in understanding cutting performance well.

## 3. Machining Difficult Materials Using Cryogenic Cooling

**Figure 8:**
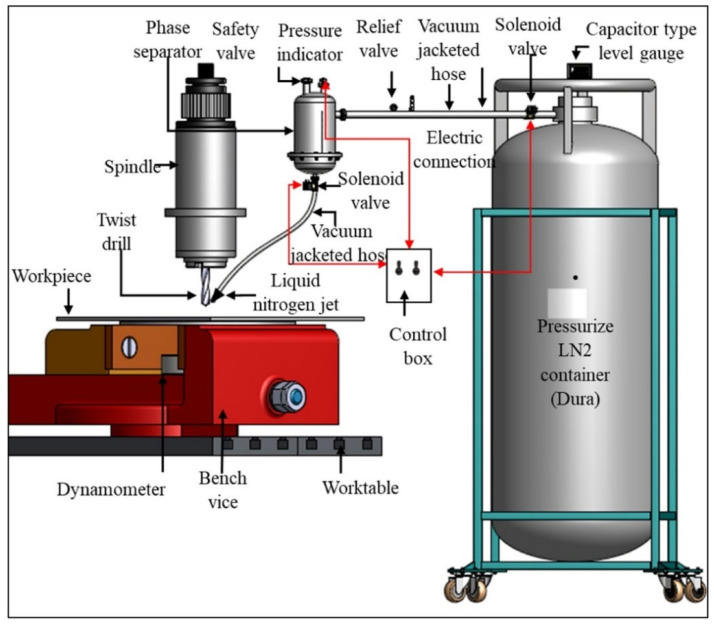
Cryogenic cooling system [97].

In order for cutting fluids to exhibit the desired performance, they must be applied correctly and effectively [93]. Cutting fluid properties, operation characteristics and requirements, and cutting tool characteristics have led to different cooling methods [94]. In order to apply cooling methods efficiently, cooling systems (tank, pump, cooling line, filter, valves, etc.) have been added to the benches as hardware. Since adding all cooling methods to the counter will increase the costs significantly, the most used cooling methods are preferred by manufacturers [95]. Cooling methods can be used directly on the machine tool or applied to the cutting area from outside. In cooling applications, care should be taken to cool the tool and workpiece, not the chip [96]. This makes the positioning of the cooling system important, as seen in Figure 8. The application pressure of the cutting fluid is effective in chip breaking, chip removal, and fluid penetration.

**Figure 9:**
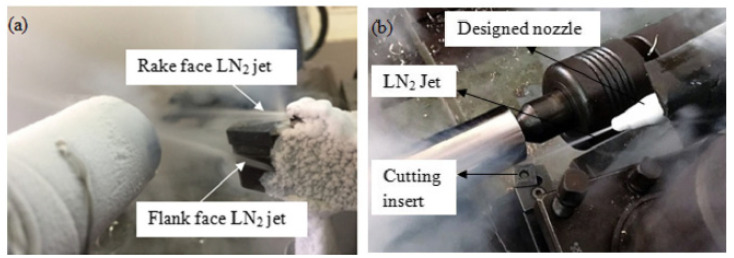
Machining zone consisting of two modes: (**a**) Mode-I, which involves a modified tool holder and tool wear, (**b**) Mode-II, which involves an external nozzle and tool wear [106].

In addition, the cryogenic coolants are supplied in the gas form. In general, the gases are a fluid that is continuous in the air shear zone [98]. The limited lubrication and cooling effects of air are effective even in dry cutting conditions. Sending compressed air to the cutting area with the help of a compressor facilitates chip evacuation, does not require any cost, has high penetration, and can enter areas where liquids cannot enter [99]. It may not be desirable for liquid residues to remain on some special materials. In such cases, the cooling effects of gases such as helium, carbon dioxide, argon, and nitrogen are used [100]. Carbon dioxide, which is compressed and sent to the cutting area, evaporates and provides cooling. In addition to systems in which air or gases are used directly, there are also applications in which they are sent to the cutting zone accompanied by other coolants and lubricants [101]. Another method of air-enhanced cooling is achieved by sending liquid nitrogen (LN_2_) along with air to the cutting area. The ability of liquid nitrogen to remain at very low temperatures (−196 °C) enabled the effective cooling of the cutting zone [102]. This effective cooling reduces tool wear by reducing the high temperatures that occur during cutting. The increase in tool life and cutting performance makes this method stand out [103]. In addition to nitrogen costs, storing nitrogen in special tanks and transporting it through special lines causes additional costs [104]. All these have led to the use of cryogenic cooling in limited areas. Królczyk et al. examined the tool life in machining of duplex stainless steel. The authors reported that as the cutting speed increased, the wear on the cutting tool edge became more intense [105]. Krolczyk et al. evaluated workpiece surface roughness, cutting force, and cutting tool life when turning duplex stainless steel under dry and cooling/lubrication conditions using three carbide cutting tools. The test results showed that dry machining and finished cutting tool quality increased cutting tool life by almost three times above cooling/lubrication conditions. They also noted that low feed rates and high cutting speeds save energy usage and improve machining efficiency [20]. Sivaiah and Chakradhar investigated the effects of cryogenic cooling conditions on cutting temperature, cutting tool flank wear, chip removal rate, surface roughness, surface topography, and micro hardness in turning 17-4 PH stainless steel. They applied cooling to the cutting zone in two different ways, as seen in Figure 9. In Mode 1, they sent LN_2_ through the cutting tool from the upper and lower points closer to the cutting tool, and in Mode 2, they used LN_2_ between the tool and the chip through an external nozzle. They stated that the Mode 1 nozzle significantly improved machining efficiency at all levels compared to the external Mode 2 nozzle in turning 17-4 PH stainless steel [106].

**Figure 10:**
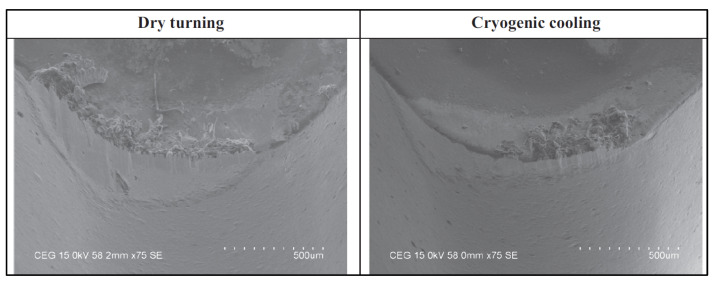
SEM images of the cutting inserts of a nickel-based superalloy [109].

**Figure 11:**
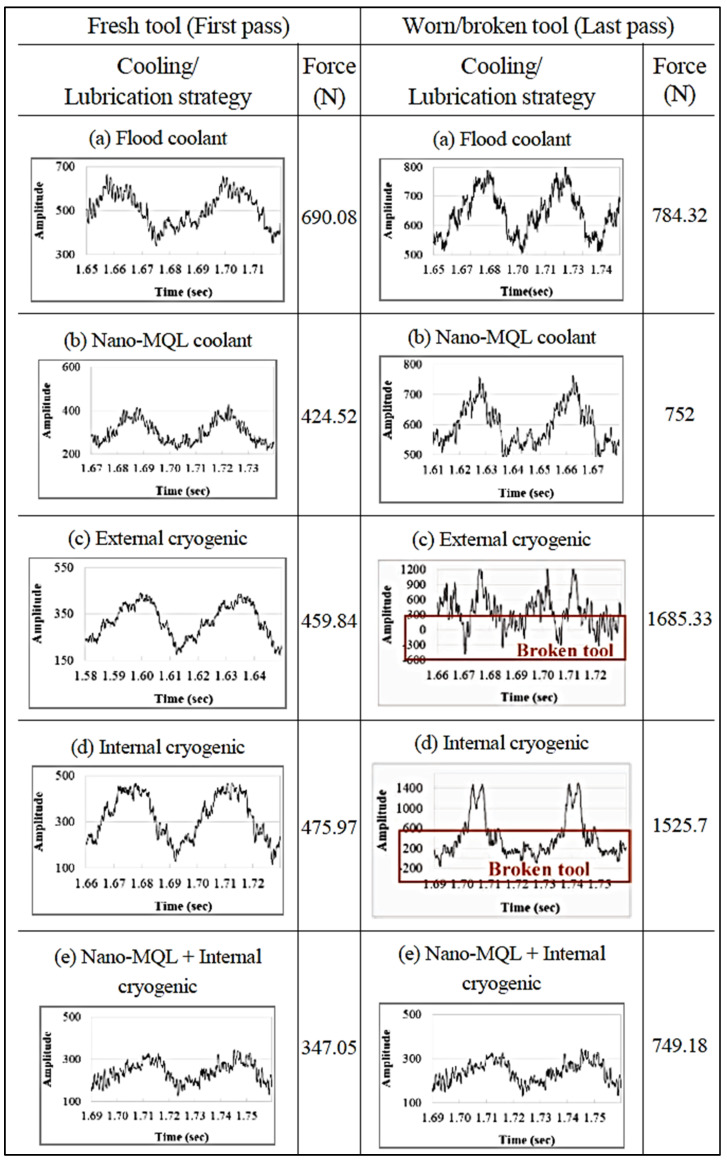
Cutting forces in the machining of Ti6Al4V under different conditions [110].

Bagherzadeh et al. examined cutting tool wear, workpiece surface roughness, cutting forces, and chip formation in the cryogenic-assisted turning of Ti6Al4V and Inconel 718 alloys. They concluded that cryogenic experiments yielded longer tool life and better workpiece surface quality than dry experiments [107]. In turning Inconel 718 super alloy, Kaynak examined the effects of dry, MQL, and cryogenic cutting conditions on cutting force, tool wear, cutting temperature, chip shape, and surface roughness. Cryogenic cooling reduced cutting zone temperature, cutting tool wear, and workpiece surface quality, according to the experiment [108]. In turning the Hastelloy C-276 super alloy material with multilayer TiAlN-coated cutting tools using PVD, Dhananchezian examined the effects of dry and cryogenic cooling on cutting temperature, cutting force, surface roughness, tool wear, and tool morphology. LN2 reduced cutting zone temperature by 61–68%, and cutting force and surface roughness by 8–33%. Cryogenic machining turned the Hastelloy C-276 material with better tool wear than dry machining, as seen in Figure 10 [109]. Park et al. evaluated tool wear and cutting forces in milling the Ti-6Al-4V titanium alloy with external, internal, and NanoMQL + internal cryogenic cooling/lubrication. The experimental results showed that NanoMQL + internal Cryo cooling conditions improve cutting forces by 51% compared to internal cryogenic cooling conditions, and significantly decrease other cutting forces (Figure 11) [110].

The impact of various cutting settings on tool wear, surface roughness, and cutting tool life in milling Ti6Al4V material under cryogenic and MQL conditions was investigated by Shokrani et al. According to their findings, cutting tool life is extended, and performance is 50% better under the cryogenic + MQL cooling/lubrication condition than under the standard cooling system [111]. Using MQL, and cryogenic conditions, Sun et al. investigated cutting force, workpiece surface roughness, and cutting tool wear in turning the Ti-5553 alloy. The experimental study found that compared to normal and MQL cooling conditions, cryogenic chilling reduced cutting force by up to 30%, improved surface roughness, and extended cutting tool life [112]. Jamil et al. examined changes in surface roughness, cutting force, and cutting temperature when turning Ti-6Al-4V under cryogenic (CO_2_) and hybrid NanoMQL (Al_2_O_3_ and MWCNT). Hybrid NanoMQL cooling reduced surface roughness by 8.72%, cutting force by 11.8%, and cutting tool life by 23% compared to cryogenic cooling. Compared to the hybrid Nano + MQL cooling condition, cryogenic cooling reduced cutting zone temperatures by 11.2% [113]. Table 1 shows the work conducted by different researchers.

## 4. Recent Developments and Modifications in Cryogenic Cooling

Lubrication and cooling applied simultaneously while machining significantly improves operational efficiency [127,128]. Cutting fluids are used to increase chip evacuation speed, reducing chip sizes, preventing chip adhesion, preventing corrosion, and increasing tool life [129]. In order for cutting fluids to have the desired effect, they must be applied correctly to the cutting area [130]. For good cooling performance, the heat conduction ability and specific heat of the cutting fluid must be high [131]. For good lubrication, the cutting fluid must have high adhesion or wetting properties [132]. Adhesion or wetting means that the fluid sticks to a surface at a certain thickness [133]. Oils, especially vegetables ones, are liquids with high adhesion ability. Properties of main cutting fluids [134]:

- High cooling ability.
- High lubrication ability.
- High corrosion protection.
- Long storage and usage life.
- Non-flammable and non-flammable properties.
- No harmful effects on health.
- Low viscosity.
- Does not adversely affect operating conditions.
- Environmentally friendly and not polluting the air and water.
- Highly recyclable.
- Does not increase costs too much.

There is no ideal cutting fluid that has all of these features [135]. Cooling and lubrication properties are the determining features for cutting fluids. Water has high heat conduction ability and specific heat. The specific heat of water is approximately twice that of oils [136]. However, the adhesion properties of water are very low [137]. Additionally, water causes corrosion, resulting in a decrease in product quality [138]. For an ideal cutting fluid, oil mixtures that have both high cooling ability and high water adhesion ability and additional substances that will contribute to this mixture are added. Cutting fluids are generally evaluated under two headings with their different properties and uses. Cutting fluids are classified as emulsion cutting fluids, chemical cutting fluids, and cutting oils [139,140]. There are conditions where gases are also used for similar purposes during cutting to increase operational efficiency. Cutting oils are used in processes where lubrication rather than cooling is important [141]. Cutting oils provide high corrosion protection and surface quality [142]. However, the risk of burning limits their use at high cutting speeds. Chemical cutting fluids consist of a mixture of water and chemicals. These liquids have superior cooling capabilities and corrosion prevention properties. Its high cooling properties provide great benefits at high cutting speeds where heat generation is high. These fluids are clear and do not obscure the operating conditions. However, its lubricating properties are very poor. Some mineral oils are added to these chemical fluids to increase their lubricating properties. These cutting fluids are called semi-chemical cutting fluids. Emulsion cutting fluids are the mixtures of water and oil [143]. The mixing ratio of water and petroleum-based mineral oil is between 5 and 10%. The mixture usually takes on a milky color. These fluids are preferred due to their high cooling, clean working environment, high corrosion prevention, low cost, low combustion, and lubrication properties. They are frequently used in all operations and cutting speeds, except very heavy metal removal operations [144].

### 4.1. Hybrid Method-1 (Cryogenic + MQL)

Studies in the literature on machining of difficult-to-machine materials generally show that, due to their frequent use in industries, machining of stainless steels [145], nickel alloys [146] and titanium alloys [147] under different cutting conditions and cutting parameters results in outputs such as surface and subsurface integrity properties, cutting forces and tool life. When a general literature research on the subject is conducted and the results obtained are compared, it can be seen that cooling applications using cryogenic liquid nitrogen (LN_2_) and cryogenic carbon dioxide gas (CO_2_) in the machining of such materials cause excessive cooling of the material at low cutting speeds and when the cutting temperature is relatively low [125]. When the studies using MQL derivatives were examined, it was observed that this method could be efficient in terms of surface roughness and cutting forces at medium cutting speeds [148], but was insufficient in cooling at high cutting speeds and increased tool wear along with the increase in force. In general, it does not seem possible to reduce the cutting forces occurring during machining in an efficient and sustainable way by using different cooling/lubrication conditions alone [149]. While there have been several studies on various strategies for lowering cutting forces in machining operations, one of the most popular is the hybrid cooling/lubrication approach, which includes cryogenic cooling [150]. In cases where cryogenic methods and MQL method derivatives are individually insufficient, it can be seen that hybrid cooling/lubrication methods, especially in machining of difficult-to-machine materials, can be quite effective in cases where the cutting speed is high [151]. Based on the findings, cryogenic methods’ cooling capabilities can lead to material hardening issues, such as increased cutting forces and surface roughness, as well as tool wear and difficulty in machining materials with low cutting speeds [152], which also occur when the MQL method and its derivatives are used at high speeds [153]. It is necessary to reduce the negative effects arising from the characteristics of the cooling/lubrication methods. To support the high cooling capacity of cryogenic methods with sufficient lubrication capacity, the good lubrication properties of the MQL method and its derivatives can be used. Therefore, in addition to the cooling/lubrication methods used, the cutting parameters and machining method must be selected together and in the most appropriate way. More research is needed in this field in order to diversify the approaches to examine cutting forces in the machining of difficult-to-machine materials to develop hybrid cooling/lubrication methods and to further clarify the combinations of cooling/lubrication, material type, parameter group, and manufacturing methods required to reduce cutting forces [154]. Table 2 listed the work on hybrid methods, especially focusing on the difficult-to-machine materials.

### 4.2. Hybrid Method-2 (Cryogenic + Nanoparticles + MQL)

**Figure 12:**
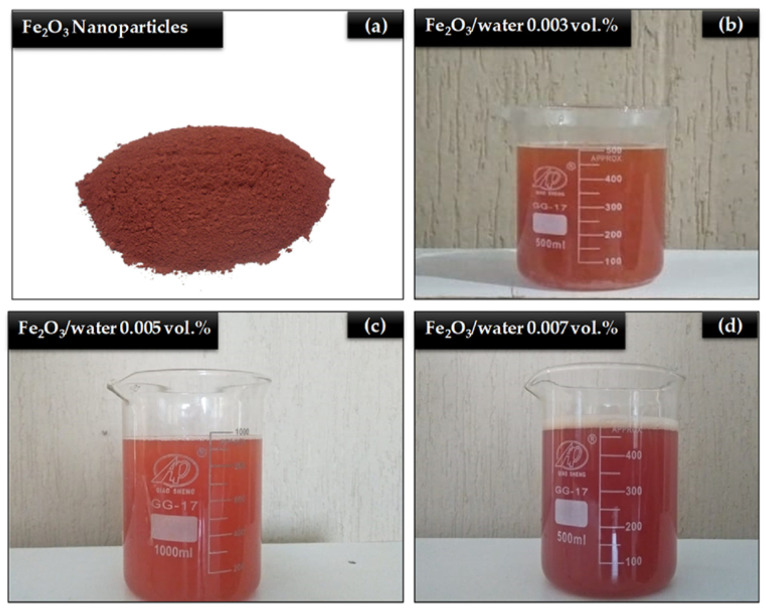
Samples of nanofluids (**a**) Ferric oxide nano powder, (**b**) nanofluid with 0.003 vol%, (**c**) nanofluid with 0.005 vol%, and (**d**) nanofluid with 0.007 vol% [164].

Nanoparticles are particles obtained by breaking down metal or non-metal materials by physical or chemical means and reducing them to nanometer size. Different nanoparticle production methods, including chemical, electrolysis, or atomization, can be used depending on the type of material and the shape and size of the desired nanoparticle. Metal oxide nanoparticles, which have good heat conduction rates and low costs, are frequently preferred as nanofluids [163]. Figure 12 shows the Fe_2_O_3_ nanoparticle with different concentrations.

**Figure 13:**
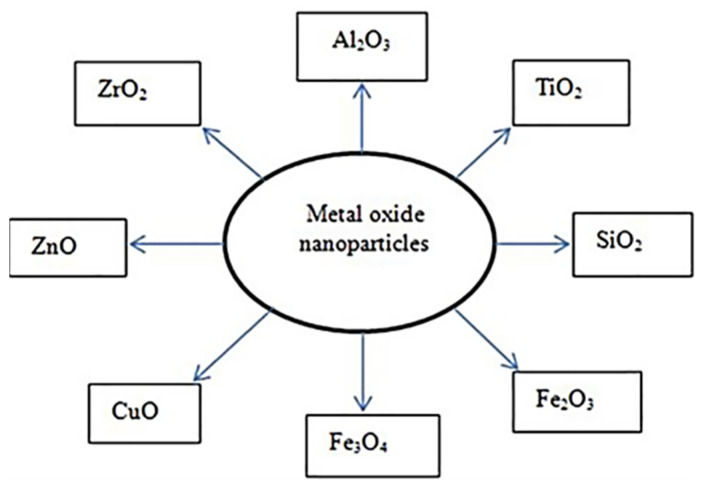
Different metal oxide nanoparticles [165].

Apart from Fe_2_O_3_, various metal oxide particles are also used to create nanofluids. There are various metal oxide nanoparticles used for different processing processes. These are aluminum oxide (Al_2_O_3_), titanium dioxide (TiO_2_), silicon dioxide (SiO_2_), iron trioxide (Fe_2_O_3_), magnetite (Fe_3_O_4_), copper oxide (CuO), zinc oxide (ZnO), and zirconium dioxide (ZrO_2_), as shown in Figure 13.

The thermal conductivity and density of nanoparticles in the base fluid determine nanofluid performance [165]. Table 3 shows metal oxide nanoparticle thermal conductivity and density.

**Figure 14:**
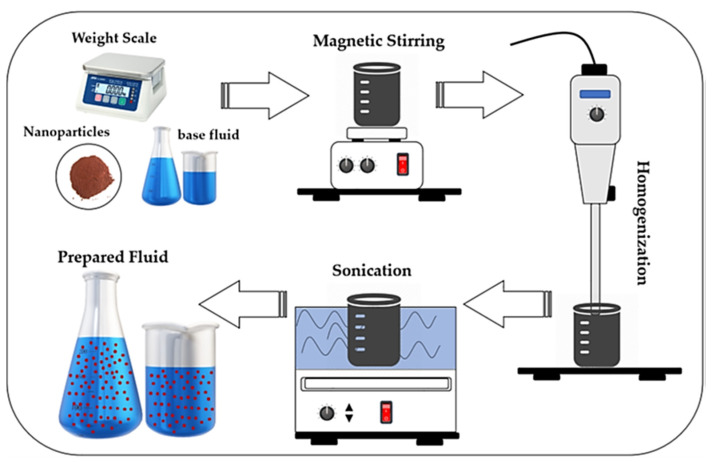
The nanofluid preparation stages [166].

Apart from metal oxides, there are various nanoparticles used for nanofluids. These are multi-walled carbon nanotubes (MWCNT), graphene, teflon (PTFE), hexagonal boron nitride (hBN), molybdenum sulfide (MoS_2_), silver (Ag), calcium fluoride (CaF_2_), and tungsten disulfide (WS_2_), which can be counted as diamonds. Some of these nanoparticles (calcium fluoride, tungsten disulfide, etc.) can be used in micro size due to their material properties. Colloid mixtures made by mixing metallic or non-metallic nanoparticles with base fluid are called nanofluids. Figure 14 shows the nanofluid preparation stages.

While preparing a nanofluid, the nanoparticle is added to the cutting fluid on a volume-based or mass-based basis, and the mixing ratio is determined. After adding the specified amount of nanoparticles to the appropriate cutting fluid, it is first subjected to magnetic stirring. Mechanical mixing is then done to obtain a homogeneous mixture. In the final stage, the mixture is also subjected to ultrasonic mixing to prevent the nanoparticles from precipitating [167]. In this way, a homogeneous and delayed collapse nanofluid is obtained. Machining performance is improved by using nanofluids in MQL systems. Nanoparticles enhance the cooling effect of cutting fluid due to their high thermal conductivity. Furthermore, cutting fluids containing nanoparticles have an improved lubricating effect due to their increased viscosity [168,169]. Furthermore, the cutting fluid’s particles have a polishing action, which further enhances surface quality [170]. Research on nanofluids’ impact on heat transfer performance has shown that the thermal conductivity of the fluid is greatly affected by the type, size, and volume of reinforced nanoparticles [171]. Table 4 shows the studies on Hybrid method-2 (Cryogenic + nanoParticles + MQL)

## 5. Conclusions and Future Scope

Ultimately, this extensive investigation into cryogenic cooling and its uses in cutting difficult-to-machine metals has exposed a new technical frontier with enormous promise for the industrial sector. The advancements discussed underscore the transformative impact of cryogenic cooling in overcoming longstanding challenges associated with the machining of difficult-to-machine alloys. This state-of-the-art review led to the following conclusions:

- Cryogenic cooling is a machining alternative. Superalloys, ferrous metal, and viscoelastic polymers/elastomers are cryogenically machined. Titanium, Inconel, and tantalum superalloys performed better with cryogenic cooling during turning, including surface roughness, tool life, tool wear, cutting forces, etc.
- Cryogenic cooling solves the main issue in machining superalloys—heat accumulated in the cutting zone due to poor thermal conductivities. If configured properly, cryogenic machining also improves ferrous metal machining. Correct cryogen application can delay/reduce ferrous steel high-speed machining tool wear and change component surface behavior. Cryogenic cooling changed hardness, adhesion, and machinability.
- Cryogenic cooling improves tool life, surface finish, and machining efficiency. The extreme temperatures of cryogenic fluids can reduce heat generation during machining. This discovery increases tool lifespan and allows for the precision cutting of previously difficult materials.
- The future of cryogenic cooling in alloy machining is bright. Further research and development can optimize cryogenic processes, explore novel cryogenic fluids, and improve system designs for efficiency and applicability. Academic-industry synergies can also advance knowledge and application.
- The adoption of cryogenic cooling is poised to expand across diverse sectors, influencing not only traditional manufacturing, but also emerging industries such as aerospace, medical devices, and electronics. The environmental sustainability of cryogenic cooling, coupled with its economic advantages, positions it as a key player in the pursuit of greener and more efficient machining practices.
- Additionally, cryogenic cooling has environmental and economic impacts. Tool wear and energy consumption reduction support sustainable manufacturing, making industry greener and more efficient. As industries seek to increase productivity and precision, cryogenic cooling shows their commitment to innovation.
- Finally, cryogenic cooling technology lays the groundwork for a dynamic precision machining age. As researchers and industry professionals explore cryogenic cooling’s possibilities, alloy machining will change, pushing innovation and excellence in manufacturing for years to come.

## Figures and Tables

**Table 1:** Studies on cryogenic cooling in the machining of difficult-to-machine materials.

Authors	Workpiece	Machining Outputs				Cooling Method
		Cutting Temperature	Cutting Force	Surface Roughness	Tool Wear	
Dhananchezian et al. [114]	Ti6Al4V	√	√	√	√	Cryogenic cooling
Iturbe et al. [115]	Inconel718			√	√	Conventional cooling and cryogenic cooling
Sartori et al. [116]	Ti6Al4V	√			√	Cryogenic cooling
Moritz et al. [117]	Ti6Al4V			√	√	Cryogenic cooling
Zhao et al. [118]	Ti6Al4V		√	√		Cryogenic cooling
Chaabani et al. [119]	Inconel718		√	√	√	Cryogenic cooling
Zurecki et al. [120]	52100 steel			√		Conventional cooling and cryogenic cooling
Aramcharoen et al. [121]	Ti6Al4V				√	Cryogenic cooling
Patel et al. [122]	Nimonic 90		√	√	√	Cryogenic cooling
Shah et al. [123]	Inconel718		√	√		Cryogenic cooling
Sivaiah et al. [124]	17-4 PH stainless steel	√	√	√	√	Conventional cooling and cryogenic cooling
Jebaraj et al. [125]	55NiCrMoV7 die steel	√		√	√	Cryogenic cooling
Gong et al. [126]	35CrMnSiA steel		√	√		Cryogenic cooling

**Table 2:** Studies on Hybrid method-1 (Cryogenic + MQL).

Authors	Workpiece	Machining Outputs			
		Cutting Force	Surface Roughness	Tool Wear/Life	Microhardness/Microstructure
Pereira et al. [155]	AISI 304 Steel	√	√		
Pusavec et al. [156]	Inconel718	√	√	√	
Pereira et al. [157]	Inconel718	√		√	
Wika et al. [158]	AISI 304 Steel		√	√	
Pereira et al. [159]	Inconel718			√	
Schoop et al. [160]	Ti6Al4V		√	√	√
Pusavec et al. [161]	Inconel718		√		
Shokrani et al. [111]	Ti6Al4V		√	√	
Pereira et al. [162]	Inconel718			√	
Gajrani et al. [153]	Ti6Al4V	√	√	√	

**Table 3:** Thermal conductivity and density properties of metal oxide nanoparticles [165].

Metal Oxide Nanoparticles	Thermal Conductivity (W/m·K)	Density (g/cm^3^)
**Al_2_O_3_**	40	3.97
**TiO_2_**	11.7	4.23
**SiO_2_**	7.6	2.4
**Fe_2_O_3_**	7	5.34
**Fe_3_O_4_**	17.65	5.18
**CuO**	29.8	6.5
**ZnO**	50	5.61
**ZrO_2_**	2	5.89

**Table 4:** Studies on Hybrid method-2 (Cryogenic + nanoParticles + MQL).

Authors	Workpiece	Machining Outputs					
		Cutting Temperature	Cutting Force	Surface Roughness	Tool Wear	Chip Morphology	Microhardness/Microstructure
Korkmaz et al. [149]	Inconel601			√	√	√	√
Sen et al. [172]	Hastelloy C276	√	√	√	√	√	√
